# RNA N6-methyladenosine reader IGF2BP2 promotes lymphatic metastasis and epithelial-mesenchymal transition of head and neck squamous carcinoma cells via stabilizing slug mRNA in an m6A-dependent manner

**DOI:** 10.1186/s13046-021-02212-1

**Published:** 2022-01-03

**Authors:** Dan Yu, Min Pan, Yanshi Li, Tao Lu, Zhihai Wang, Chuan Liu, Guohua Hu

**Affiliations:** Department of Otorhinolaryngology, The First Affiliated Hospital of Chongqing Medical University, No. 1 Youyi Road, Yuzhong District, Chongqing, 400016 China

**Keywords:** N6-methyladenosine, IGF2BP2, Slug, EMT, Lymphatic metastasis, HNSCC

## Abstract

**Background:**

Lymph node metastasis is the main cause of poor prognosis of head and neck squamous carcinoma (HNSCC) patients. N6-methyladenosine (m6A) RNA modification is an emerging epigenetic regulatory mechanism for gene expression, and as a novel m6A reader protein, IGF2BP2 has been implicated in tumor progression and metastasis. However, not much is currently known about the functional roles of IGF2BP2 in HNSCC, and whether IGF2BP2 regulates lymphatic metastasis through m6A modification in HNSCC remains to be determined.

**Methods:**

The expression and overall survival (OS) probability of m6A-related regulators in HNSCC were analyzed with The Cancer Genome Atlas (TCGA) dataset and GEPIA website tool, respectively. The expression levels of IGF2BP2 were measured in HNSCC tissues and normal adjacent tissues. To study the effects of IGF2BP2 on HNSCC cell metastasis in vitro and in vivo, gain- and loss- of function methods were employed. RIP, MeRIP, luciferase reporter and mRNA stability assays were performed to explore the epigenetic mechanism of IGF2BP2 in HNSCC.

**Results:**

We investigated 20 m6A-related regulators in HNSCC and discovered that only the overexpression of IGF2BP2 was associated with a poor OS probability and an independent prognostic factor for HNSCC patients. Additionally, we demonstrated that IGF2BP2 was overexpressed in HNSCC tissues, and significantly correlated to lymphatic metastasis and poor prognosis. Functional studies have shown that IGF2BP2 promotes both HNSCC cell migration as well as invasion via the epithelial-mesenchymal transition (EMT) process in vitro, and IGF2BP2 knockdown significantly inhibited lymphatic metastasis and lymphangiogenesis in vivo. Mechanistic investigations revealed that Slug, a key EMT-related transcriptional factor, is the direct target of IGF2BP2, and essential for IGF2BP2-regulated EMT and metastasis in HNSCC. Furthermore, we demonstrated that IGF2BP2 recognizes and binds the m6A site in the coding sequence (CDS) region of Slug and promotes its mRNA stability.

**Conclusions:**

Collectively, our study uncovers the oncogenic role and potential mechanism of IGF2BP2, which serves as a m6A reader, in controlling lymphatic metastasis and EMT in HNSCC, suggesting that IGF2BP2 may act as a therapeutic target and prognostic biomarker for HNSCC patients with metastasis.

**Supplementary Information:**

The online version contains supplementary material available at 10.1186/s13046-021-02212-1.

## Background

Head and neck squamous cell carcinoma (HNSCC), which can develop in the larynx, pharynx, and oral cavity, ranks as the sixth leading malignancy worldwide, resulting in approximately 350,000 deaths each year [1, 2]. The majority of newly diagnosed patients with HNSCC present at the locally advanced stages and the majority of these patients have regional lymph node (LN) metastasis at the onset [2, 3]. Despite recent advances in therapeutic approaches, such as multidisciplinary treatment and targeted immunotherapy [4–7], the 5-year overall survival (OS) rate of HNSCC patients is still only~ 50%, mainly owing to the high rate of lymphatic metastasis and postoperative recurrence [2–4]. Therefore, clarifying the molecular mechanisms that underlie HNSCC initiation and metastasis is of great importance for the improvement in patient prognosis and development of targeted therapeutic strategies.

An RNA binding protein (RBP) known as the insulin-like growth factor 2 mRNA-binding protein 2 (IGF2BP2) is related to the regulation of various cellular and biological processes [8, 9]. Abnormal expression of IGF2BP2 is commonly linked to multiple diseases, including cancers [10, 11]. Accumulating evidence has revealed the oncogenic role of IGF2BP2 in tumorigenesis and metastasis [12–15]. Experiments with IGF2BP2-deficient mice has indicated that IGF2BP2 is a tumor-promoting factor that facilitates the progression of cancer as well as metastasis [16]. Meanwhile, IGF2BP2 overexpression induces genome instability and promotes cancer cell proliferation and migration in vitro [13, 17, 18]. It has been demonstrated recently that proteins of the IGF2BP family (IGF2BPs, including IGF2BP2) are a distinct type of N^6^-methyladenosine (m6A) readers, which are capable of regulating and recognizing the m6A modification of target mRNAs and crucial for their oncogenic functions [19].

Among the hundreds of internal RNA modifications, m6A is considered to be the most prevalent type in eukaryotic mRNA [20–22]. The m6A modification is reversible, and is catalysed by methyltransferases (“writers”) and removed by demethylases (“erasers”) [20, 23]. In this dynamic process, the fate and biological function of m6A-modified RNAs mainly relies on the m6A binding proteins (“readers”) [23, 24]. “Readers” are those that can recognize and bind directly to m6A modification sites, and exert more specific regulatory functions, including alternative splicing, stability of mRNA, RNA processing, and translation [25, 26]. IGF2BPs and the YT521-B homology (YTH) domain family (YTHDF) are the two crucial RBP families of m6A “readers” [26, 27]. Different from YTHDF that may accelerate mRNA decay [27], IGF2BPs, as newly identified m6A readers, can target numerous mRNAs and maintain their stability to promote cancer progression and metastasis in a m6A-dependent manner [28, 29]. Several recent studies have reported that m6A modification is implicated in lymphatic metastasis in cancers [30–32]. However, the functional roles of IGF2BP2 in HNSCC, and more specifically whether IGF2BP2 regulates lymphatic metastasis in HNSCC by modifying m6A is currently unclear.

The primary cause of over 90% of mortality related to cancer is tumor metastasis [33, 34], and epithelial–mesenchymal transition (EMT) has been proven to be the initial crucial factor in tumor metastasis [35]. The process of EMT is characterized by the loss of cell adhesion and epithelial markers, like E-cadherin, which is a fundamental event for EMT; and results in the gain of enhanced invasive capacities and mesenchymal markers, including N-cadherin and vimentin [36]. EMT is mediated by a variety of EMT transcriptional factors (EMT-TFs), including Snail, Slug, ZEB1, and Twist, which can inhibit the expression of E-cadherin to promote the motility and invasiveness of cancer cells [37, 38]. Slug, encoded by the SNAI2 gene, is a key transcriptional factor for EMT that orchestrates a variety of biological processes critical to tumorigenesis and metastasis [39, 40]. Several studies have demonstrated that Slug can be regulated by RNA binding proteins at the post-transcriptional level, promoting the mesenchymal cell properties and metastasis of tumor cells [41–43]. However, whether IGF2BP2 regulates Slug expression via m6A modification and affects the process of EMT in cancer cells still needs to be elucidated.

In this study, we report a direct correlation between increased expression of IGF2BP2 with lymphatic metastasis and decreased survival of HNSCC patients. Our results show that knockdown of IGF2BP2 resulted in a significant reduction in lymphatic metastasis and lymphangiogenesis in a popliteal LN metastasis model. Mechanistically, we identified that Slug is the direct target mRNA of IGF2BP2 and is critical to the EMT process and metastasis in HNSCC. More importantly, we demonstrated that IGF2BP2 recognizes and binds the m6A site in the coding sequence (CDS) of Slug and stabilizes its mRNA.

## Methods

### TCGA data mining and analysis tools

RNA-sequencing data (level 3) in raw form and related clinical data regarding HNSCC were derived online from The Cancer Genome Atlas (TCGA) dataset (https://portal.gdc.cancer.gov/), which contained 502 HNSCC cases and 44 normal cases. Analysis and visualization of the genes’ expression was conducted with R software (version 4.0.3) packages ‘ggplot2’ and ‘pheatmap’. The GEPIA website tool (http://gepia2.cancer-pku.cn/#index) was used to conduct the OS analysis. Analysis and visualization of the Kaplan-Meier survival analysis was performed with the R software packages ‘survival’ and ‘survminer’. Multivariate cox regression analysis was conducted in order to determine the fitting terms, which were used for building the nomogram. The ‘forestplot’ package, found in R, was used to identify each variable’s *P* value, hazard ratio (HR), and 95% confidence interval (CI). To predict the 1-, 3-, and 5-year overall recurrence, we developed a nomogram in conformity with the results of the multivariate Cox proportional hazards analysis. The nomogram is a visual representation of factors, and can be used for calculating the risk of recurrence for a distinct patient through summing up the points correlated to each risk factor in the ‘rms’ package of R. The two-gene correlation map was constructed with the ‘ggstatsplot’ package in R software based on Spearman’s correlation analysis. SRAMP (http://www.cuilab.cn/sramp/), a motif-dependent m6A site predictor, was used to predict m6A modification sites on the RNA sequences of interests.

### HNSCC patients and clinical samples

For this study, 20 paired fresh HNSCC tissues and normal adjacent tissues (NATs), and 20 fresh HNSCC tissues with or without lymphatic metastasis (consisting of 10 cases with lymphatic metastasis and 10 cases without lymphatic metastasis) were retrieved from HNSCC patients who received surgery at the Department of Otorhinolaryngology of the First Affiliated Hospital of Chongqing Medical University (Chongqing, China). Fresh tissues were washed with saline after resection, and liquid nitrogen was used to freeze them immediately after which they were kept at − 80 °C for total protein and RNA extraction. Furthermore, in total 78 HNSCC tissue specimens embedded in paraffin were collected from the Department of Pathology of the First Affiliated Hospital of Chongqing Medical University. The 78 HNSCC patients underwent surgery between January 2012 and December 2019 in the Otolaryngology department of the First Affiliated Hospital of Chongqing Medical University. The clinical features of the patients are detailed in Table 1. Informed consent was acquired from all patients before surgery. The study was authorized by the Biomedical Ethics Committee of the First Affiliated Hospital of Chongqing Medical University.

**Table 1:** Association between IGF2BP2 expression and clinicopathological characteristics of patients with HNSCC

Characteristics	Total	IGF2BP2 expression		𝜒2	***P*** Value
		High	Low		
**Age(y)**				1.192	0.275
**≥60**	48	26	22		
**<60**	30	20	10		
**Gender**				0.448	0.504
**Male**	74	43	31		
**Female**	4	3	1		
**T classification**				1.184	0.757
**T1**	5	4	1		
**T2**	13	8	5		
**T3**	36	21	15		
**T4**	24	13	11		
**N classification**				8.804	0.032^*^
**N0**	27	10	17		
**N1**	10	6	4		
**N2**	33	24	9		
**N3**	8	6	2		
**Tumor Differentiation**				2.096	0.351
**Well**	19	12	7		
**Moderate**	39	20	19		
**Poor**	20	14	6		
**Lymph node metastasis**				8.214	0.004^*^
**No**	27	10	17		
**Yes**	51	36	15		
**Extranodal extension**				0.115	0.734
**No**	67	39	28		
**Yes**	11	7	4		

*Abbreviations*: *IGF2BP2* Insulin-like growth factor 2 mRNA-binding protein 2, *HNSCC* Head and neck squamous carcinoma cells

The *P* Value was measured by Chi-square test. *P < 0.05

### Cell lines and cell culture

FaDu cells were obtained from the Chinese Academy of sciences (Shanghai, China). SCC15 cells were the kind gift of Prof. Kai Yang (Chongqing Medical University, China). FaDu and SCC15 cells were both kept in Dulbecco’s modified Eagle’s medium (DMEM, Gibico, USA), to which 10% fetal bovine serum (FBS, PAN-Biotech, Germany) and 1% penicillin-streptomycin was added, in an atmosphere of 37 °C and 5% CO_2._

### Immunohistochemistry (IHC) staining and scoring analyses

IHC was performed with a detection kit (SP-9000, Beijing, Zhongshan Jinqiao) in accordance with the manufacturer’s instructions. In short, fresh xylene was used to deparaffinize the paraffin sections, which were then hydrated in gradient alcohol. The citric acid buffer was used for antigen retrieval at 90 °C–100 °C for 30 min to which an appropriate amount of endogenous peroxidase blocker was added. The standard goat serum working solution was sealed at room temperature for 15 min, and then transferred to a refrigerator of 4 °C and incubated with IGF2BP2 and LYVE-1 primary antibodies overnight. On the second day, horseradish enzyme-labeled streptavidin was dripped into the working solution, then incubated for 15 min at room temperature, and rinsed with PBS 3 times for 3 min each time. Diaminobenzidine (DAB) was used for color development, followed by hematoxylin staining for 30 s, and rinsing with tap water for 5 min. Finally, it was dehydrated, transparent and mounted with neutral gum. The IHC stained sections were reviewed and scored independently by two superior pathologists. A final score was then calculated by multiplying the signaling intensity score and the staining distribution score. The signal intensity scores were categorized as follows: 0 (no signal), 1 (weak), 2 (moderate), and 3 (strong). The staining distribution scores were determined according to the percentage of positive cells: 0 (0–5%), 1 (5–25%), 2 (25–50%), 3 (50–75%), 4 (75–100%). The median value was chosen as the cutoff.

### Extraction of RNA and quantitative real-time PCR (qRT-PCR)

An E.Z.N.A.^®^ Total RNA Kit I (Omega Bio-tek, USA) was utilized for isolation of the total RNA from HNSCC specimens and cells according to instructions of the manufacturer. Subsequently, an Agilent 2100 Bioanalyzer (Agilent, Santa Clara, CA, USA) was used to determine the total RNA’s quality through absorbance readings at 260 nm. Reverse transcription of total RNA into complementary DNA (cDNA) was done with a gDNA Eraser (TaKaRa, Japan) using a PrimerScript™ RT Reagent Kit. Next, for the amplification process an ABI 7500 Real-Time PCR System (Applied Biosystems, Foster City, CA, USA) and a SYBR Premix Ex Tag™ Kit (TaKaRa, Japan) were utilized in accordance with instructions of the manufacturer. The conditions in which the PCR amplification was conducted was as follows: 40 cycles in total at 95 °C for the duration of 30 s, then 95 °C for 5 s, and lastly 60 °C for 1 min. Glyceraldehyde 3-phosphate dehydrogenase (GAPDH) functioned as internal control. The 2^-ΔΔCt^ method was applied for analysis of the results, which were presented as relative expression. All experiments were performed three times to independently verify the findings. The primers for PCR analysis are listed in Additional file 1: Table S1.

### Protein extraction and western blotting analysis

Total proteins from HNSCC specimens and cells were derived with a protein extraction kit (KGP250, KeyGen, Jiangsu, China). A bicinchoninic acid (BCA) protein assay kit (P0010S, Beyotime, Shanghai, China) was utilized for the detection of the concentration of protein. Next, protein extracts were diluted in 5 × loading buffer and boiled for 10 min. The boiled proteins were subjected to 10% SDS-PAGE, placed onto polyvinylidene fluoride (PVDF) membranes, and blocked with 5% nonfat dry milk for the duration of 2 h. Subsequently, we incubated the membranes overnight at 4 °C with primary antibodies, of which the details are shown in Additional file 2: Table S1. The next day, horseradish peroxidase (HRP)-conjugated secondary antibodies were used for incubating the membranes for 1 h in total at room temperature. Finally, an enhanced chemiluminescence (ECL) kit (12043-D10, Advansta, USA) was utilized to visualize the protein blots, of which the images were taken with the ChemiDoc Touch Imaging System (Bio-Rad, USA) and analyzed by ImageJ software (version v1.8.0).

### siRNA and cell transfection

To reduce the off-target effect of the siRNAs, three independent siRNAs targeting IGF2BP2 and a negative control siRNA were constructed and generated by GenePharma (Shanghai, China). In brief, HNSCC cells were seeded, at a concentration of 2 × 10^5^ cells/well, in six-well plates with culture medium, and incubated till 60%–70% confluence. Then, Lipofectamine iMAX Reagent (Invitrogen, USA) was utilized for transfection of the cells with siRNAs at a ratio of 1:3 and incubated for 24 h in serum-free medium. 24 h later, we discarded the transfection medium, and fresh complete culture medium without siRNA and Lipofectamine iMAX was added. At the timeframe of 48 h or 72 h following transfection, we collected the cells for protein or RNA extraction to conduct RT-qPCR and western blotting to validate the transfection efficiency. The IGF2BP2 target and negative control sequences are listed in Additional file 1: Table S1.

### Lentivirus vector and cell infection

For knockdown, short hairpin RNA (shRNA) of human IGF2BP2 was cloned into a hU6-MCS-Ubiquitin-firefly_Luciferase-IRES-puromycin lentiviral vector (GV344, Genechem, Shanghai, China). To achieve overexpression, human full-length cDNA of IGF2BP2 was cloned into a Ubi-MCS-3FLAG-CBh-gcGFP-IRES-puromycin lentiviral vector (GV492, Genechem, Shanghai, China). The target sequences of IGF2BP2 have been detailed in Additional file 1: Table S1. When HNSCC cells had reached the logarithmic growth phase, the cells were seeded in a six-well plate at a concentration of 5 × 10^4^ cells/well and incubated until roughly 30% confluence was reached. Then, the cells were infected with shRNA or overexpression lentiviral vectors with HiTransG A infection-enhancing solution according to the multiplicity of infection (MOI, MOI = 10). After incubating for 16 h at 37 °C, the viral medium was discarded and substituted by fresh culture. Then, the cells were screened in culture medium containing puromycin (2 μg/ml) for 1 week and stably silenced- and overexpressed-IGF2BP2 HNSCC cells were generated.

### Wound-healing and Transwell assays

Wound-healing and transwell assays were carried out in vitro for the detection of cell migration and invasion. The detailed procedures of these assays have been described in our previous study [44].

### Popliteal lymphatic metastasis model in vivo

Male BALB/cA nude mice aged 4–6 weeks and with a weight between 18 and 22 g were obtained from Huafukang Biotechnology Co., (Beijing, China), and accommodated in specific pathogen free (SPF) barrier facilities. To construct the metastasis model, 5 × 10^6^ FaDu cells were transfected with sh-IGF2BP2-luc and sh-NC-luc, suspended in 60 μl PBS, and then injected into the footpads of the mice. Six weeks after injection, mice were subjected to bioluminescence imaging to evaluate lymphatic metastasis. For bioluminescence imaging, mice were anesthetized by inhaling 2% isoflurane for approximately 5 min, injected intraperitoneally with D-Luciferin potassium salt (200 μl, 150 μg/ml, ST196, Beyotime, Shanghai, China), and imaged with a bioluminescence system (NightOwl II LB983, Berthold Technologies, Germany). All the primary tumors and popliteal LNs were harvested and embedded in paraffin for IHC analysis. The LN volumes were calculated based on this formula: LN volume (mm^3^) = length × width^2^ × 0.5. All the experiments were authorized by the Laboratory Animal Use Management Committee of the Experimental Animal Center of Chongqing Medical University.

### Immunofluorescence

FaDu and SCC15 cells seeded on sterile coverslips were fixated with 4% paraformaldehyde for the duration of 20 min, then permeabilized with 0.1% Triton X-100 for 15 min, and lastly blocked with goat serum at room temperature for a total of 30 min. Subsequently, primary antibodies were utilized for incubation of the cells overnight at 4 °C, of which the details are shown in Additional file 2: Table S1. The following day, fluorescence-labeled secondary antibodies were utilized incubation of the cells for 1 h at room temperature. DAPI (C1006, Beyotime, Shanghai, China) was used to stain nuclear DNA and the images were captured with a confocal system (Nikon).

### RNA binding protein immunoprecipitation (RIP)

HNSCC cells at 90% confluence, cultured in 10-cm plates (approximately 1.2 × 10^7^ cells each plate), were accumulated and then lysed with IP lysis buffer (P0013J, Beyotime, Shanghai, China) supplemented with protease inhibitor (100×) and RNase inhibitor (40 U/μl) on ice for the duration of 30 min. The cell lysates, pipetted up and down several times, were stored in − 80 °C for 5 min, then allowed to thaw on ice, and centrifuging at 12,000 g for 10 min. Then the cell lysates were divided into two parts, one was saved as input group for the whole cell extraction, and the other was used for the following immunoprecipitation (IP) treatment (IP group). For the IP group, cell lysates were incubated with 5 μg anti-IGF2BP2 (ab128175, Abcam, USA) or IgG antibody (14678–1-AP, Proteintech, Wuhan, China), respectively, which was alternated continually throughout the night at 4 °C. Protein A/G magnetic beads (Bimake, China) were rinsed five consecutive times with 0.1% Tween-20 in PBS, and then mixed with cell lysate-antibody complexes and rotated continuously at 4 °C for 6 h. Next, the RNA-protein complexes were rinsed five consecutive times with elution buffer (containing 150 mM NaCl, 50 mM Tris-HCl, 5 mM EDTA, 0.5 mM DTT, 0.5% NP-40, 10% SDS, and RNase inhibitor) and treated with proteinase K at 55 °C for 1 h. Bound RNAs were extracted and subjected to RT-qPCR for quantitative analysis. Relative enrichment was normalized to the input. The primers for RT-qPCR were showed in Additional file 1: Table S1.

### MeRIP-qPCR

Total RNA was extracted as described above. Ten percent of the total RNA was reserved for the input control and the remaining RNAs were used for m6A-IP. Anti-m6A antibody (ab151230, abcam, USA) or mouse IgG were immobilized on magnetic beads using the Dynabeads™ Antibody Coupling Kit (14311D, Invitrogen, USA) according to the manufacturer’s instruction. Subsequently, total RNA was incubated with antibody-conjugated beads in 500 μl binding buffer (containing 140 mM NaCl, 50 mM Tris-HCl, 5 mM EDTA, 0.5% NP-40, and RNase inhibitor) and rotated continually for 4 h at 4 °C. M6A-modified mRNAs were eluted from the beads with elution buffer and then purification was carried out for further analysis by RT-qPCR. Relative enrichment was normalized to the input. The primers for RT-qPCR were showed in Additional file 1: Table S1.

### Luciferase reporter assay

2 × 10^5^ HNSCC cells were first seeded in plates with 24-wells, followed by culturing for 24 h. Then plasmids carrying wild-type or mutated-type Slug CDS were transfected into the cells. After transfection for 12 h, cells were re-seeded into a plate with 96-wells and then for 24 h incubated. The Dual-Luciferase^®^ Reporter Assay System (E1910, Promega, USA) was used to analyze the luciferase activities. Renilla Luciferase (R-luc) was used to achieve normalization of the firefly luciferase (F-luc) activity.

### mRNA stability assay

HNSCC cells were seeded in plates with six-wells and grown to approximately 50% confluence following incubation for 24 h. Then, actinomycin D (5 μg/ml, Sigma, USA) was used to treat the cells, which were harvested at 0 h, 3 h, and 6 h. The total RNA was derived and then analyzed with qRT-PCR. The mRNA value of each group was calculated and normalized to GAPDH at the designated time. The degradation rate of mRNA was approximated based on previously published protocols [45].

### Statistical analysis

All the statistical analysis were conducted with GraphPad Prism (version 7.0, GraphPad software, USA) and SPSS 21.0 software (IBM, SPSS Statistics, USA). The correlation between IGF2BP2 expression and clinicopathologic parameters were analyzed with the Chi-Square test. Univariate and multivariate regression analyses were employed with the Cox proportional hazards model to identify independent factors affecting the survival of HNSCC patients. The Kaplan-Meier method was carried out to produce survival curves, and any significant differences in survival probability between groups were compared by log-rank statistics. We applied the two-tailed Student’s *t* test to compare results between two different groups, and the one-way ANOVA was conducted for multiple comparisons. All the data are presented in the form of mean ± SD and are the result of no less than 3 independently performed experiments. Statistical significance was determined as values of *P* < 0.05. *P < 0.05, **P < 0.01, ***P < 0.001, ****P < 0.0001, ns: not statistically significant.

## Results

### Expression and clinical significance of m6A regulators in HNSCC

**Fig. 1:**
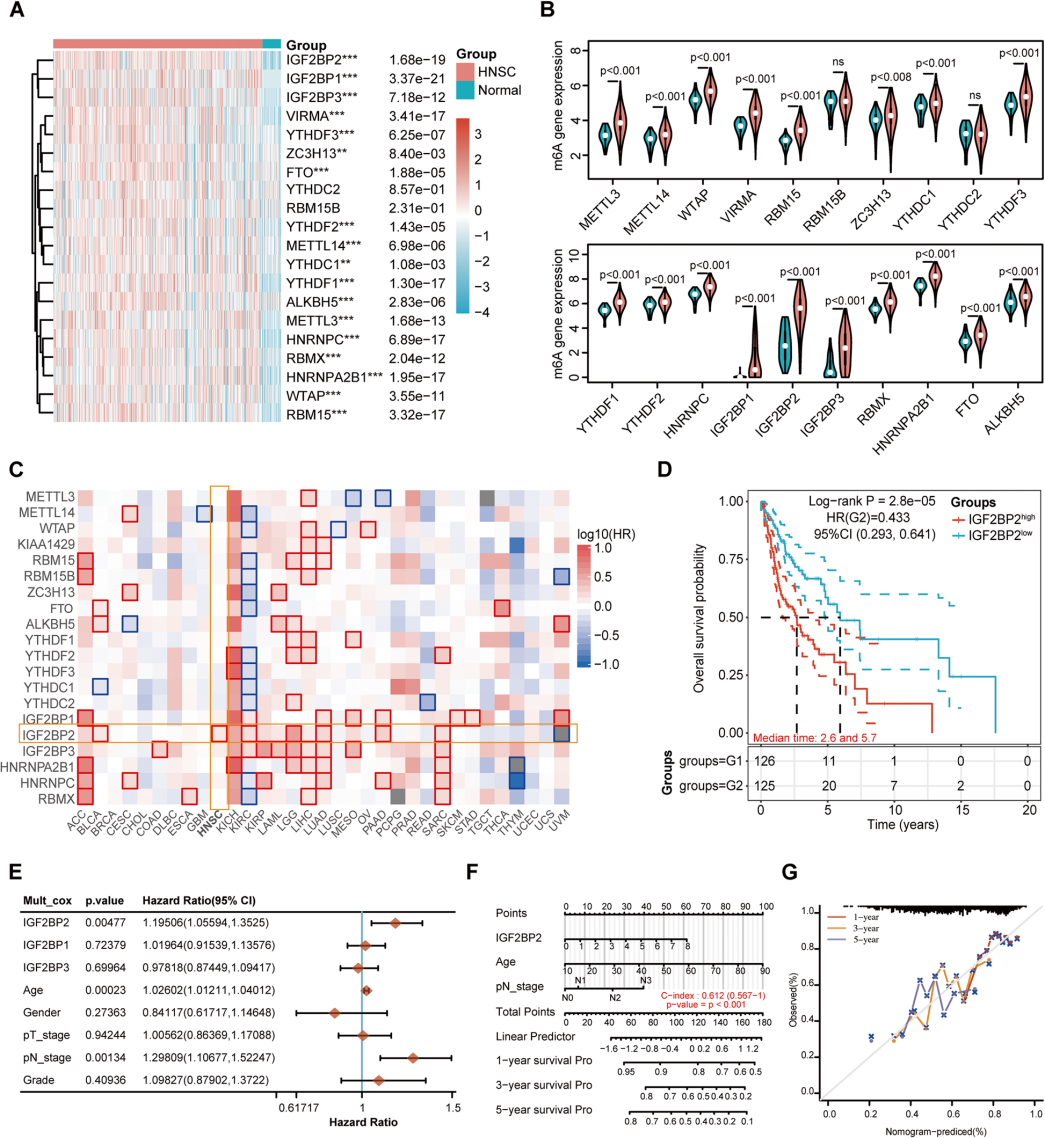
Expression and clinical significance of m6A regulators in HNSCC. (**A** and **B**) Heatmap and violin plots show the mRNA expression levels of 20 m6A regulators in 502 HNSCC and 44 normal tissues according to TCGA data. Red and blue colors illustrate relatively high and low expression, respectively. **P < 0.01, ***P < 0.001, ns: not statistically significant. **C** The survival map of 20 m6A-related regulators in pan-cancer generated by GEPIA2 online website. **D** The OS probability of HNSCC patients with high vs. low expression of IGF2BP2. The quartile expression level of IGF2BP2 was used as cutoff value. Group 1: the upper quartile was considered as IGF2BP2 ^high^. Group 2: the lower quartile was considered as IGF2BP2 ^low^. **E** Multivariate cox regression analysis of IGF2BPs and clinical parameters to identify the independent prognostic factors. **F** The nomogram for prediction of the 1-, 3-, and 5-year OS of HNSCC patients. **G** The calibration curve for the OS nomogram model. The ideal nomogram is represented by a dashed diagonal line, and the blue, red, and orange lines symbolize the 1-, 3-, and 5-year observed nomograms

We analyzed the expression profiles of 20 m6A-related regulators in 502 HNSCC and 44 normal tissues from the TCGA database in order to study the role of m6A modification in HNSCC. As shown in Fig. 1A and B, 18 of the 20 m6A regulatory genes were significantly upregulated in HNSCC tissues compared with normal tissues, including 6 “writers” (VIRMA, ZC3H13, METTL14, METTL3, WTAP, and RBM15), 2 “erasers” (FTO and ALKBH5), and 10 “readers” (IGF2BP2, IGF2BP1, IGF2BP3, YTHDF3, YTHDF2, YTHDC1, YTHDF1, HNRNPC, RBMX, and HNRNPA2B1). There was no statistically significant difference between the expression levels of YTHDC2 and RBM15B in HNSCC tissues and normal tissues. To further investigate the clinical association of the m6A-related regulators in HNSCC, we first analyzed the OS rate of the 20 m6A regulatory genes in pan-cancer using GEPIA2. Intriguingly, among all these m6A-related genes, only the higher expression of IGF2BP2 is associated with a lower survival probability in HNSCC patients (Fig. 1C). The Kaplan-Meier survival analysis based on TCGA data also supported this result, suggesting a potential prognostic role of IGF2BP2 in HNSCC (Fig. 1D). The multivariate cox regression analysis revealed that IGF2BP2 was an independent risk factor in HNSCC patients (Fig. 1E). In addition, the pathological N stage was also a significantly independent prognostic factor, suggesting that lymphatic metastasis is closely related to a poor prognosis in HNSCC patients (Fig. 1E). Furthermore, we constructed a nomogram in accordance with the results of the multivariate cox regression analysis, in which the three independent prognostic factors, including IGF2BP2, pathological N stage and age were combined to produce a clinically quantitative method for predicting the 1-, 3-, and 5-year survival probability of HNSCC patients (Fig. 1F). Every patient would accumulate a number of points for each prognostic parameter, and the more higher the total number of points, the poorer the outcome is for that particular patient [46]. The results of the calibration curve indicated a sufficient efficiency in estimating the 1-, 3-, and 5-year survival probability by using the nomogram (Fig. 1G). Collectively, m6A regulators were commonly overexpressed in HNSCC, and IGF2BP2 was identified as a potential prognostic biomarker in HNSCC. Therefore, we focused on IGF2BP2 for further investigation.

### High expression of IGF2BP2 in HNSCC tissues and related to lymphatic metastasis

**Fig. 2:**
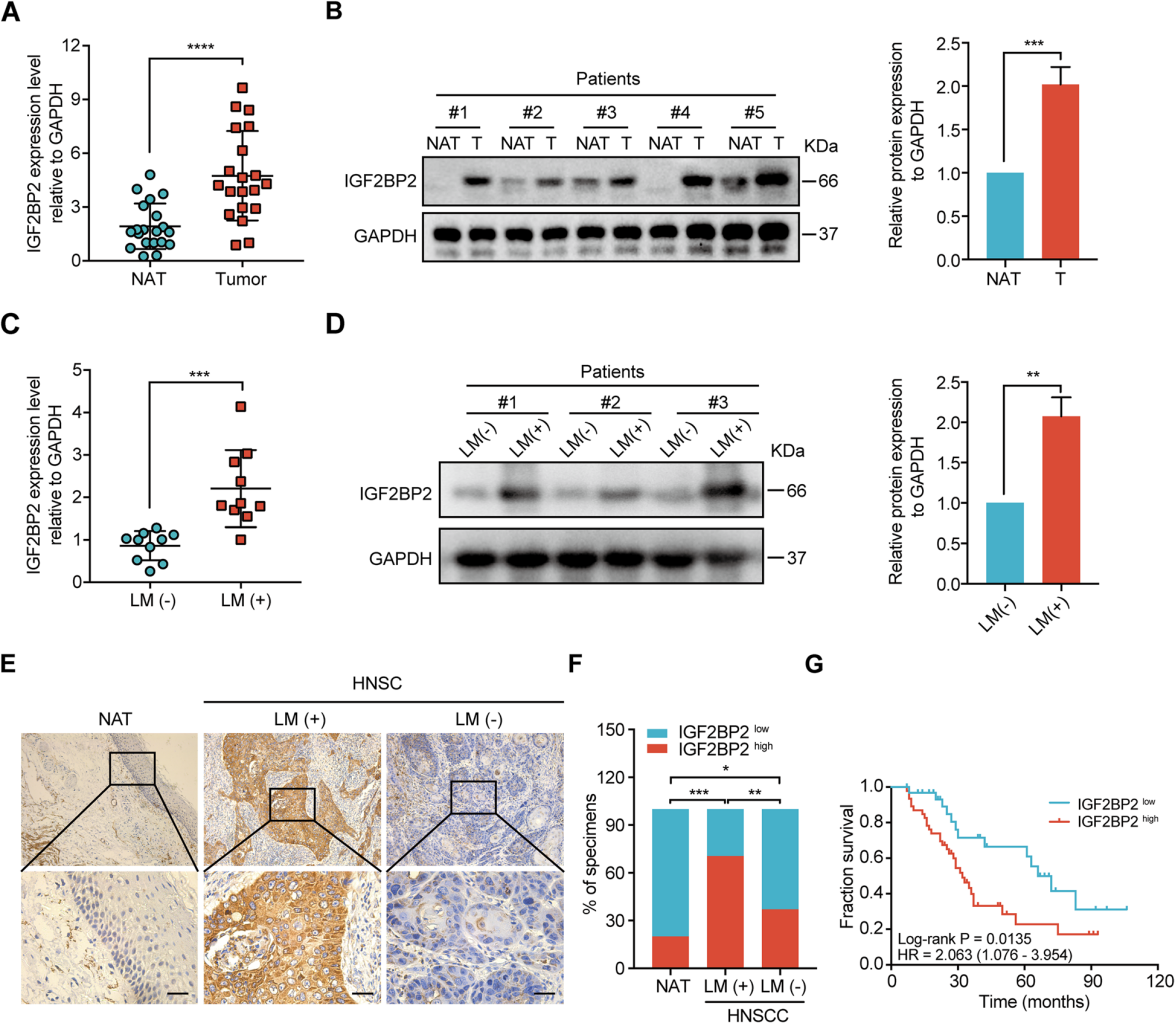
IGF2BP2 highly expressed in HNSCC tissues and related to lymphatic metastasis. **A** RT-qPCR analysis of the expression of IGF2BP2 in HNSCC tissues (*n* = 20) and NATs (*n* = 20). NAT: normal adjacent tissues; T: tumor. ****P < 0.0001. **B** Western blot analysis of IGF2BP2 expression in HNSCC tissues and NATs (*n* = 5). Right panel shows the statistical data. ***P < 0.001. **C** RT-qPCR analysis of the expression of IGF2BP2 in HNSCC tissues with or without lymphatic metastasis (*n* = 20, including 10 cases with lymphatic metastasis and 10 cases without lymphatic metastasis). LM (+): lymphatic metastasis; LM (−): non-lymphatic metastasis. ***P < 0.001. **D** Western blot analysis of the expression of IGF2BP2 in HNSCC tissues with or without lymphatic metastasis. Statistical data is shown in the right panel. **P < 0.01. **E-F** Representative images (**E**) and percentages (**F**) of IGF2BP2 expression in the paraffin-embedded HNSCC tissues with or without lymphatic metastasis and NATs. Scale bars: 200 μm. *P < 0.05, **P < 0.01, ***P < 0.001. **G** Kaplan-Meier curves of OS in HNSCC patients with high and low expression of IGF2BP2. The median IGF2BP2 expression was applied as the cutoff value. GAPDH served as an internal control. All the data are presented in the form of mean ± SD from three independently performed experiments

We initially examined IGF2BP2’s expression in HNSCC and NATs with RT-qPCR and western blot to evaluate its role in HNSCC. The RT-qPCR analysis demonstrated that IGF2BP2 was significantly overexpressed in HNSCC tumor samples compared with NATs (Fig. 2A). Similar results were observed with western blot analysis in 5 cases of HNSCC tumor and NATs (Fig. 2B). Our previous work has shown that cervical LN metastasis was correlated with the poor prognosis of HNSCC patients and was an independent risk factor for survival [47]. According to the results of the Kaplan-Meier survival analysis in Fig. 1C and D, we wondered whether IGF2BP2 is involved in lymphatic metastasis, leading to a poor prognosis in HNSCC patients. To verify this hypothesis, 20 cases of HNSCC tissues with or without lymphatic metastasis (10 cases with lymphatic metastasis and 10 cases without lymphatic metastasis) were collected for assessing IGF2BP2 expression with RT-qPCR and western blot, respectively. Their results indicated that both the mRNA as well as protein levels of IGF2BP2 were increased in HNSCC tissues with lymphatic metastasis compared with those without lymphatic metastasis (Fig. 2C and D). To further investigate the clinical relevance of IGF2BP2 in HNSCC, IHC was performed to detect IGF2BP2 expression in 78 cases of HNSCC patients, of which the complete clinicopathological characteristics and data on follow-up were available. Consistent with our data derived from RT-qPCR and western blotting, IHC analysis revealed that IGF2BP2 expression was marginally detected in NATs and slightly increased in HNSCC tissues without lymphatic metastasis but strongly upregulated in those with lymphatic metastasis (Fig. 2E and F). Importantly, high IGF2BP2 expression was associated with poor OS probability (Fig. 2G). Moreover, analysis of the clinicopathological characteristics demonstrated that IGF2BP2 expression was significantly correlated with the pathological N classification and LN metastasis (Table 1). Univariate and multivariate Cox regression analyses demonstrated that IGF2BP2 expression was an independent prognostic factor in patients with HNSCC (Table 2). Collectively, these results imply that there is a high expression of IGF2BP2 in HNSCC and that it has an important role to play in LN metastasis.

**Table 2:** Univariate analysis and multivariate analysis of IGF2BP2 expression and clinicopathologic variables in patients with HNSCC Cox-regression analysis

Variables	Univariate analysis		Multivariate analysis	
	HR (95%CI)	***P*** Value	HR (95%CI)	***P*** Value
IGF2BP2	2.254 (1.160-4.381)	0.017*	2.206 (1.065-4.568)	0.033*
Age	1.006 (0.545-1.857)	0.983	1.154 (0.594-2.244)	0.672
Gender	0.607 (0.145-2.538)	0.494	0.469 (0.101-2.172)	0.333
T classification	1.157 (0.554-2.416)	0.698	1.087 (0.496-2.382)	0.836
Tumor differentiation	1.164 (0.608-2.226)	0.647	1.049 (0.528-2.084)	0.892
LNM	2.915 (1.295-6.559)	0.010*	2.555 (1.082-6.033)	0.032*
Extranodal extension	0.704 (0.296-1.678)	0.429	0.978 (0.065-1.568)	0.653

*Abbreviations*: *IGF2BP2* Insulin-like growth factor 2 mRNA-binding protein 2, *HNSCC* Head and neck squamous carcinoma cells, *HR* Hazard ratio, *CI* Confidence interval, *LNM* Lymph node metastasis.

Variables: IGF2BP2: high vs low; Age: ≥60 (y) vs<60 (y); Gender: male vs female; T classification: T1/T2 vs T3/T4; Tumor differentiation: well/moderate vs poor; LNM: N0 vs N1/N2/N3; Extranodal extension: no vs yes. *P < 0.05.

### IGF2BP2 promotes metastatic behaviour of HNSCC cells in vitro

**Fig. 3:**
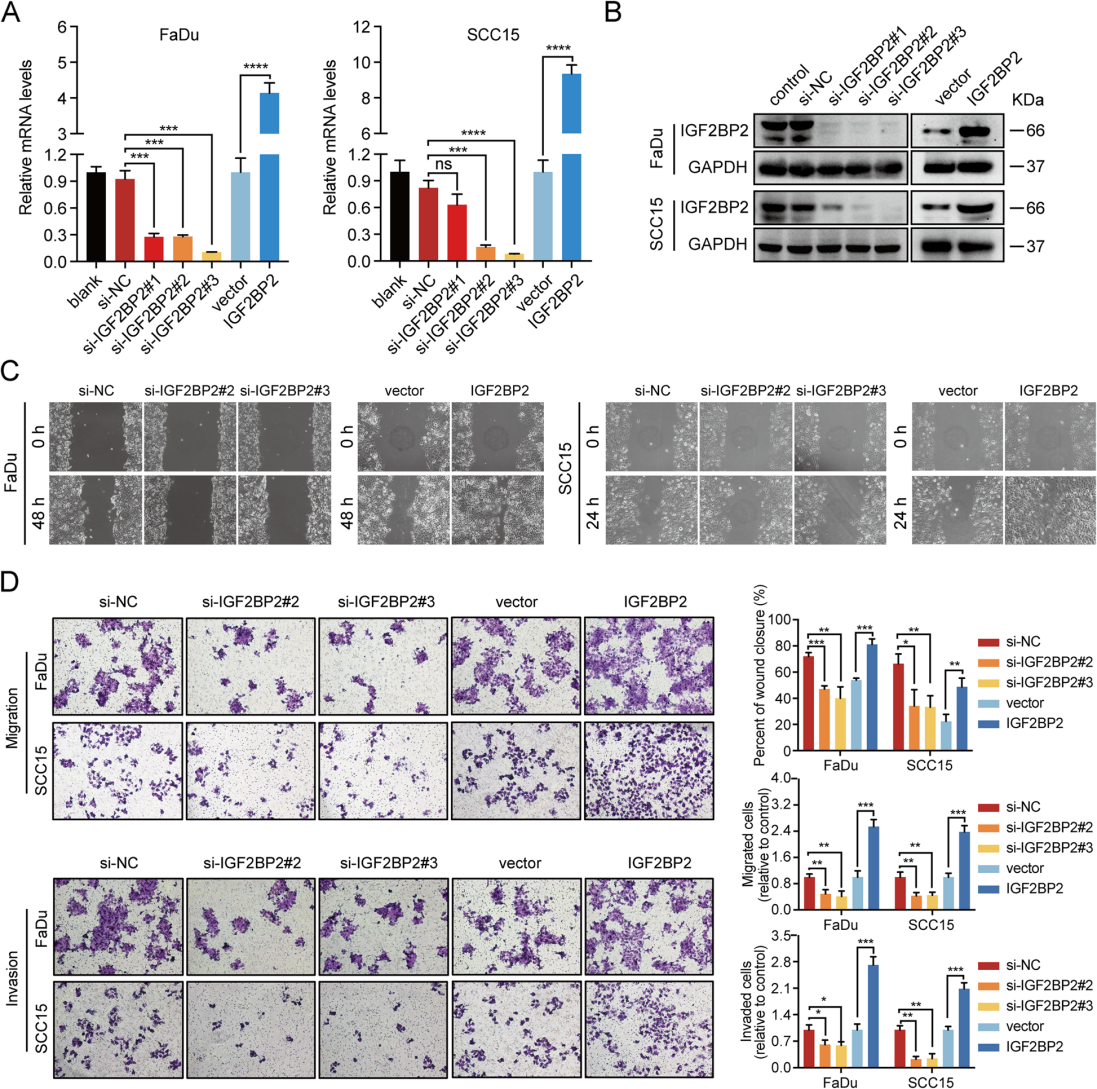
IGF2BP2 promotes metastatic behaviour of HNSCC cells in vitro. **A-B** The efficiency of knockdown and overexpression of IGF2BP2 was validated by RT-qPCR (A) and western blot (**B**) at mRNA as well as protein levels in FaDu and SCC15 cells. GAPDH served as an internal control. ***P < 0.001, ****P < 0.0001, ns: not statistically significant. **C-D** Images representing wound healing (**C**) and transwell assays (**D**) showing the mobility and invasiveness of HNSCC cells after knockdown or overexpression of IGF2BP2. Lower right panel shows the statistical data of cell migration distance, and migrated and invaded cell counts. *P < 0.05, **P < 0.01, ***P < 0.001. All the data are presented in the form of mean ± SD from three independently performed experiments

LN metastasis is a complex process involving multiple alterations, including an enhanced ability in cell migration and invasion [48, 49]. To clarify whether IGF2BP2 induces the migration and invasion ability of HNSCC cells, FaDu and SCC15 HNSCC cell lines were transfected with three small interference sequences targeting different sites (si-IGF2BP2#1, si-IGF2BP2#2, and si-IGF2BP2#3) of IGF2BP2 or were established to stably overexpress IGF2BP2 by lentivirus. The efficiency of knockdown and overexpression was validated by RT-qPCR and western blot, and the results indicated that IGF2BP2 was successfully silenced (si-IGF2BP2#2 and si-IGF2BP2#3) or overexpressed at the mRNA as well as protein levels in FaDu and SCC15 cells (Fig. 3A and B). Moreover, a wound-healing analysis demonstrated that knockdown of IGF2BP2 inhibited the migratory ability of FaDu and SCC15 cells, whereas the opposite results were observed after IGF2BP2 overexpression (Fig. 3C and D). Transwell analysis further confirmed these results by showing that silencing IGF2BP2 attenuated the mobility and invasiveness of FaDu and SCC15 cells, whereas IGF2BP2 overexpression enhanced the cell metastasis. Collectively, these data indicate that IGF2BP2 promotes the migration and invasiveness of HNSCC cells.

### IGF2BP2 knockdown suppresses lymphatic metastasis and lymphangiogenesis in vivo

**Fig. 4:**
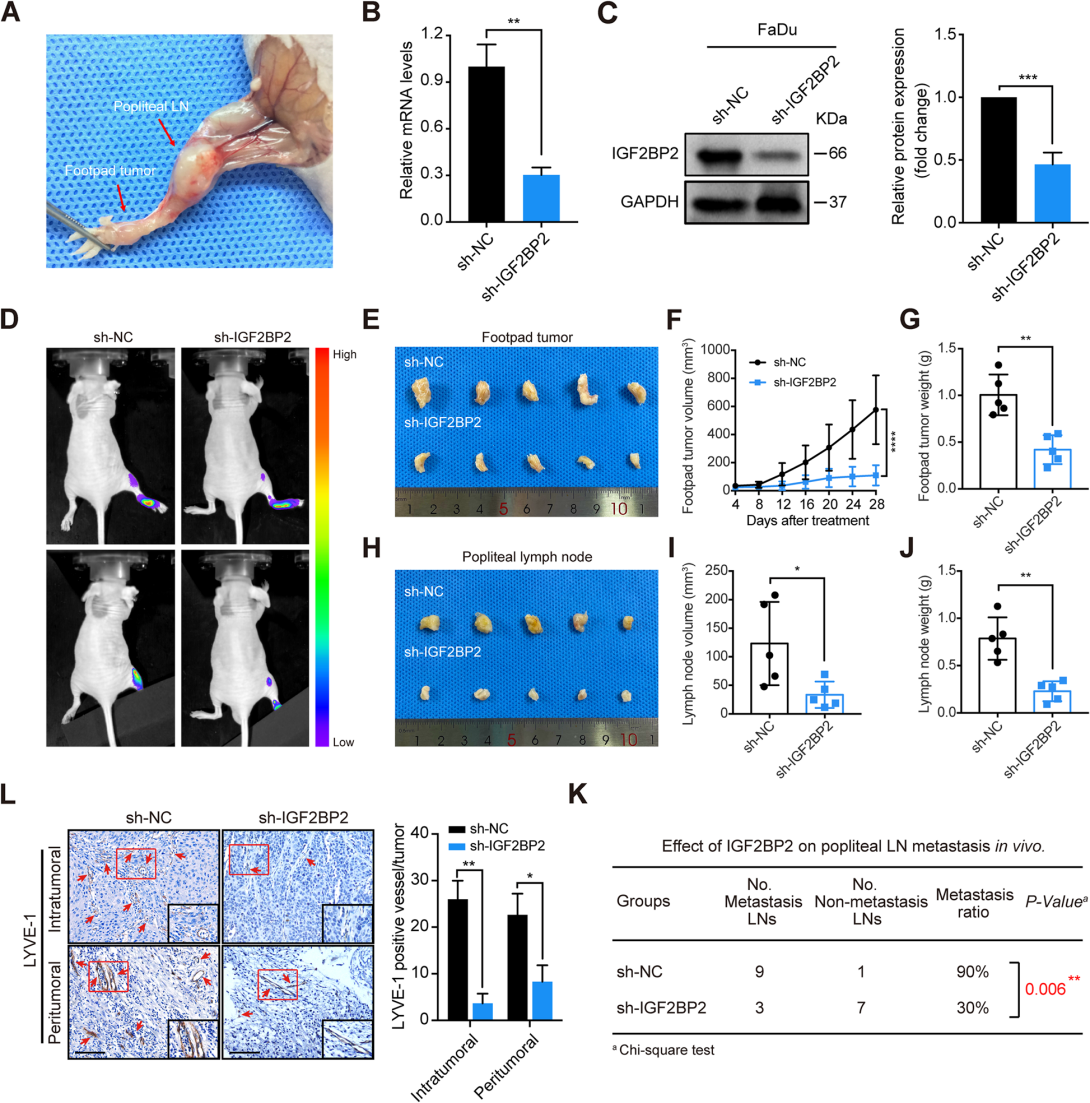
IGF2BP2 knockdown suppresses lymphatic metastasis and lymphangiogenesis in vivo. **A** Representative images of the popliteal lymph node (LN) metastasis model in nude mice. FaDu cells were inserted into the footpads of nude mice through injection, and the metastasized popliteal LNs were harvested and analyzed. **B-C** RT-qPCR (**B**) and western blot (**C**) analysis of IGF2BP2 expression at mRNA as well as protein levels in stably IGF2BP2-silenced cells and control cells. Right panel shows the statistical analysis of western blotting. GAPDH functioned as internal control. **P < 0.01, ***P < 0.001. **D** Representative images of bioluminescence of popliteal LN metastasis after inhibition of IGF2BP2 (*n* = 10 per group). **E** Representative images of enucleated footpad tumors for the indicated groups (*n* = 10 per group). **F-G** The volume (**F**) and weight (**G**) of footpad tumors for the indicated groups. **P < 0.01, ****P < 0.0001. **H** Images representing enucleated popliteal LNs for the indicated groups (*n* = 10 per group). **I-J** The volume (**I**) and weight (**J**) of lymph nodes for the relevant groups. *P < 0.05, **P < 0.01. **K** The metastatic ratio of popliteal LNs was calculated for all groups (*n* = 10 per group, *P* = 0.006). **L** Representative images of mice tissues immunostained with anti-LYVE-1 antibody in intratumoral or peritumoral regions with different IGF2BP2 expression levels. Red arrows indicate the microlymphatic vessels. Right panel shows the quantification of microlymphatic vessel density. Scale bars: 200 μm. *P < 0.05, **P < 0.01. All the data are presented in the form of mean ± SD from three independently performed experiments

To further determine the role of IGF2BP2 in lymphatic metastasis of HNSCC, we used nude mice to establish a popliteal LN metastasis model (Fig. 4A), as mentioned by a previous study [50]. In brief, FaDu cells were transfected with lentiviral vector expressing shRNA and firefly luciferase to stably silence IGF2BP2 expression, and the silencing efficiency of IGF2BP2 was verified by RT-qPCR and western blot (Fig. 4B and C). Then these cells were inserted into the footpads of nude mice through injection. After 4 weeks, the impact of IGF2BP2 on lymphatic metastasis was determined by in vivo bioluminescence imaging. Strikingly, IGF2BP2 knockdown notably inhibited the lymphatic metastasis of HNSCC cells (Fig. 4D). Moreover, the footpad tumors and the popliteal lymph nodes were both smaller and lighter in the sh-IGF2BP2 group than in the sh-NC group, suggesting that IGF2BP2 suppressed the tumorigenesis and lymphatic metastasis of HNSCC (Fig. 4E-J). In addition, the sh-IGF2BP2 group exhibited a lower LN metastatic rate than the sh-NC group (Fig. 4K). Importantly, IHC analysis showed that silencing IGF2BP2 significantly decreased the levels of microlymphatic vessel density (MLD) in both the intratumoral and peritumoral regions of mice tissues, as indicated by LYVE1-positive microvessels (Fig. 4L). Collectively, these findings suggest that IGF2BP2 knockdown significantly inhibits lymphatic metastasis and lymphangiogenesis in vivo.

### IGF2BP2 regulates EMT program of HNSCC cells

**Fig. 5:**
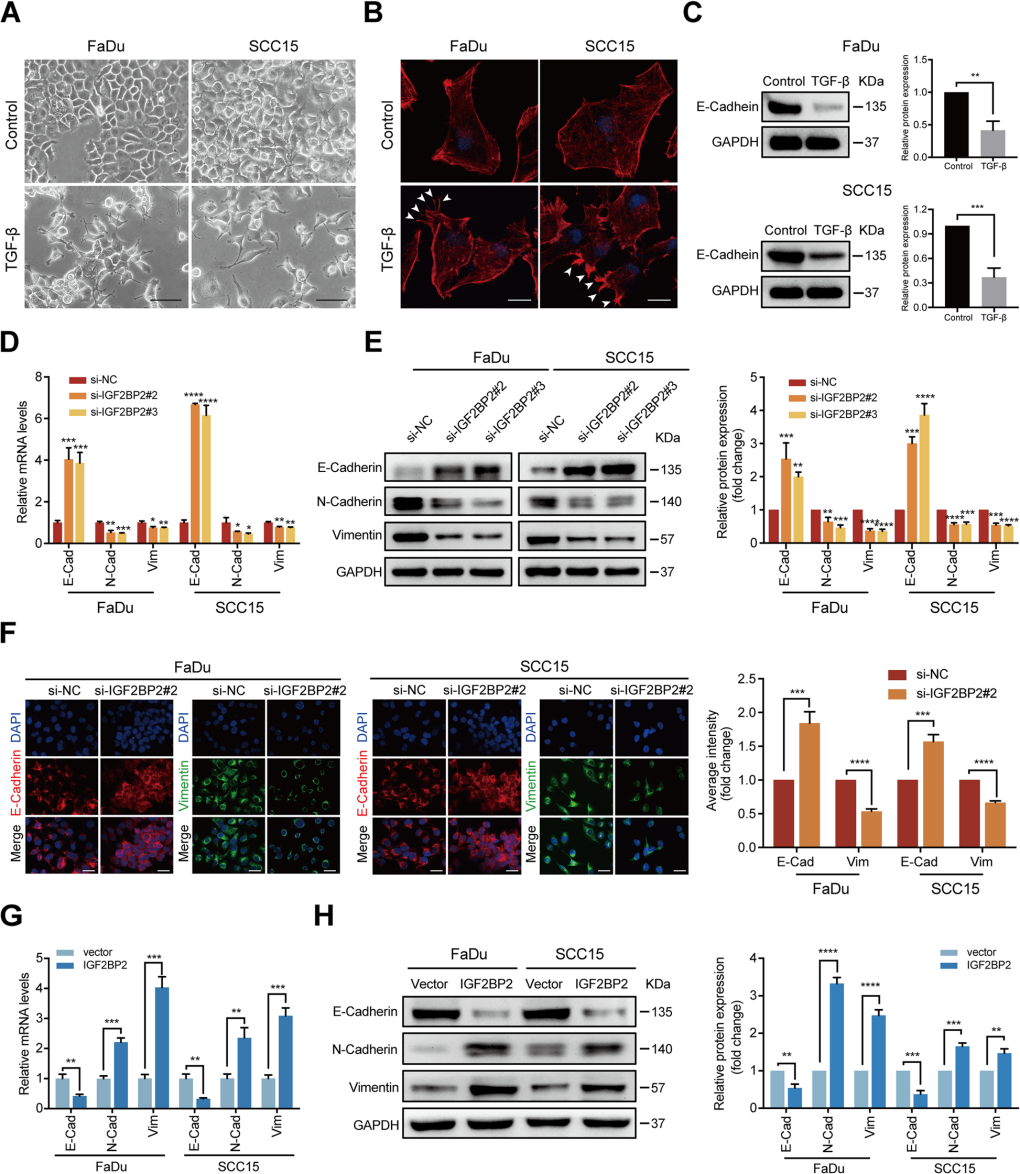
IGF2BP2 regulates EMT program of HNSCC cells. **A** FaDu and SCC15 cells were either treated with TGF-β (10 ng/ml) or without. Cell morphologic changes were observed after 72 h. Representative images from 3 independent experiments are shown. Scale bars: 200 μm. **B** FaDu and SCC15 cells were either treated with TGF-β or without. Cytoskeleton alterations were assessed by immunofluorescence staining with phalloidin, and by confocal scanning after 72 h. White arrows indicate the extensive filopodia and lamellipodia. Representative images from 3 independent experiments are shown. Scale bars: 50 μm. **C** The results of the western blot analysis of the expression of E-Cadherin in FaDu and SCC15 cells either treated with TGF-β or without. Right panel shows the statistical analysis of western blot. **P < 0.01, ***P < 0.001. **D-E** FaDu and SCC15 cells underwent transfection with si-RNAs or si-NC after TGF-β treatment. The levels of E-Cadherin, N-Cadherin, and vimentin expression were identified with RT-qPCR (**D**) and western blotting (**E**). *P < 0.05, **P < 0.01, ***P < 0.001, ****P < 0.0001. **F** FaDu and SCC15 cells were transfected with si-RNAs or si-NC after TGF-β treatment. The expression and distribution of E-Cadherin and vimentin was identified by immunofluorescence and confocal scanning. Red: E-Cadherin; Green: vimentin; Blue (DAPI): nuclei. Scale bars: 100 μm. Right panel shows the statistical analysis of immunofluorescence. ***P < 0.001, ****P < 0.0001. **G-H** FaDu and SCC15 cells were transduced with IGF2BP2 and a corresponding control vector, without TGF-β treatment. The expression levels of E-Cadherin, N-Cadherin, and vimentin were identified with RT-qPCR (**G**) and western blotting (**H**). **P < 0.01, ***P < 0.001, ****P < 0.0001. All the data are presented in the form of mean ± SD from three independently performed experiments

EMT is a critical step in the initiation of tumor metastasis [35]. To further investigate the mechanism behind the lymphatic metastasis mediated by IGF2BP2, we established a EMT cell model using TGF-β, a potent EMT inducer [51], to analyze what role IGF2BP2 plays in regulating EMT. First, 10 ng/ml TGF-β was used to treat FaDu and SCC15 cells. After 72 h treatment, we observed loss of cell-cell contact and a spindle-shape morphology characterized by mesenchymal cells (Fig. 5A). Furthermore, immunofluorescent staining and confocal imaging analysis showed that FaDu and SCC15 cells treated with TGF-β formed extensive filopodia and lamellipodia, as indicated by white arrows, which enabled cells to acquire migratory and invasive capabilities (Fig. 5B). Moreover, western blot analysis revealed that TGF-β induced the reduction of E-Cadherin expression in both HNSCC cells, which is considered a hallmark of an EMT shift (Fig. 5C). These data suggests that HNSCC cell lines treated with TGF-β were in the process of EMT.

Subsequently, we determined the role of IGF2BP2 in HNSCC cells undergoing EMT. RT-qPCR and western blot analyses showed that IGF2BP2 depletion downregulated N-Cadherin and vimentin’s expression levels, whereas that of E-Cadherin was upregulated at the mRNA as well as protein levels in FaDu and SCC15 cells treated with TGF-β (Fig. 5D and E). Moreover, immunofluorescent staining and confocal imaging analysis further confirmed that silencing IGF2BP2 led to a loss of vimentin expression, concomitant with a retention of E-Cadherin on the cell surface in both HNSCC cells, suggesting IGF2BP2 may regulate the EMT program (Fig. 5F). To further confirm the role of IGF2BP2 in EMT, we detected the EMT-related markers in FaDu and SCC15 cells transduced with IGF2BP2 and the corresponding control vector without TGF-β treatment, and observed that E-Cadherin was indeed downregulated, whereas N-Cadherin and vimentin were upregulated at both the mRNA as well as protein levels (Fig. 5G and H). Taken together, these findings suggest that IGF2BP2 has an essential function in regulating the EMT program in HNSCC cells.

### Slug is involved in IGF2BP2-regulated EMT in HNSCC cells

**Fig. 6:**
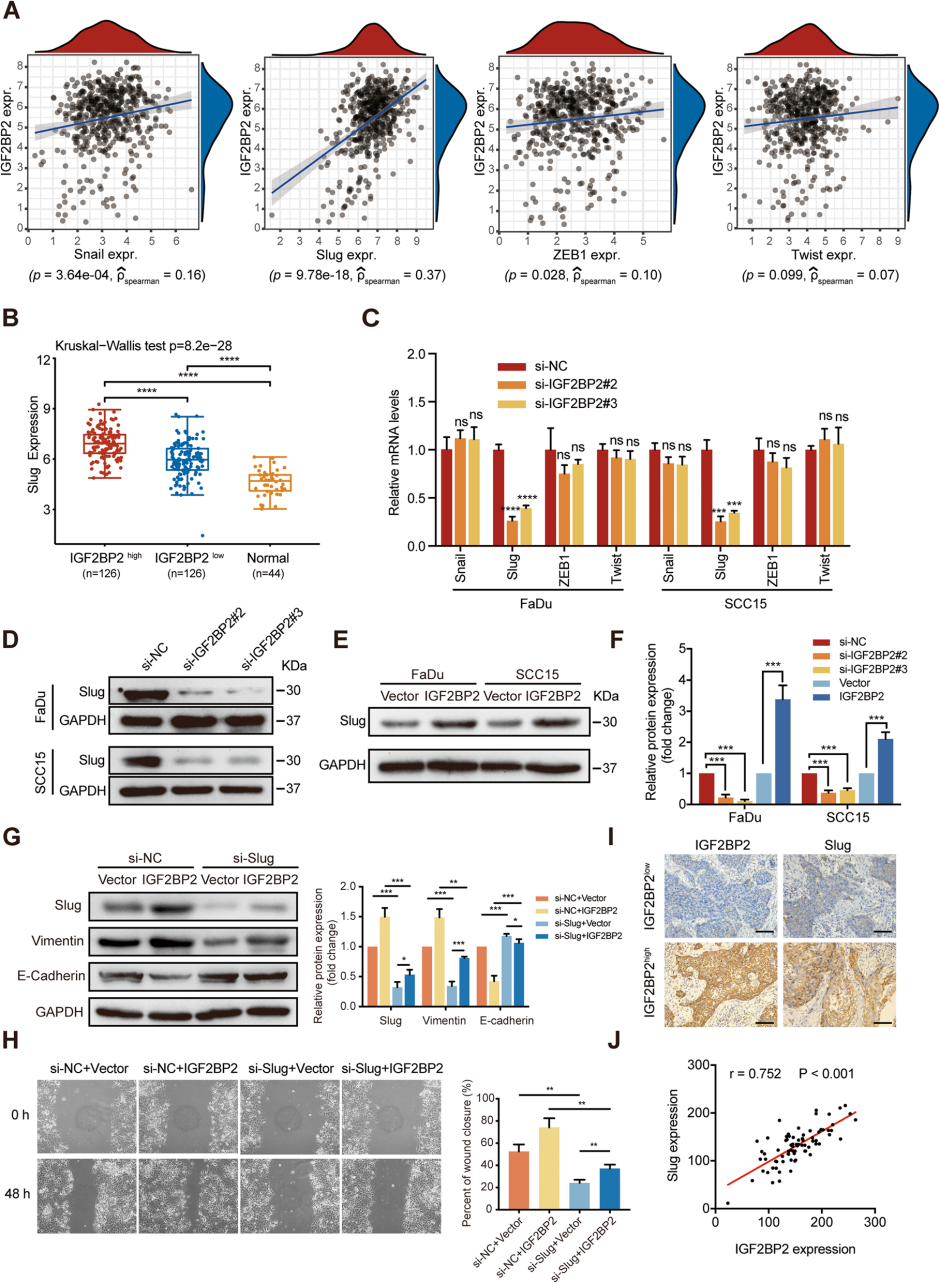
Slug is involved in IGF2BP2-regulated EMT in HNSCC cells. **A** Spearman correlation of Snail, Slug, ZEB1, and Twist with IGF2BP2 in the TCGA database for HNSCC. *P* value and Spearman’s correlations (rho) are indicated. **B** The distribution of Slug expression in HNSCC tissues with high or low IGF2BP2 expression and normal tissues. The quartile expression level of IGF2BP2 was used as cutoff value. The upper quartile was considered as IGF2BP2 ^high^, and the lower quartile was considered as IGF2BP2 ^low^. The uppermost corner on the left depicts the test method for significant *P* values. **C** FaDu and SCC15 cells were transfected with si-RNAs or si-NC after TGF-β treatment. The mRNA levels of Snail, Slug, ZEB1, and Twist were detected by RT-qPCR. ***P < 0.001, ****P < 0.0001, ns: not statistically significant. **D** FaDu and SCC15 cells underwent transfection with si-RNAs or si-NC after TGF-β treatment. The expression of Slug was detected by western blot analysis. **E** The expression of Slug was detected by western blotting analysis in IGF2BP2-overexpressed FaDu cells or corresponding control cells without TGF-β treatment. **F** Statistical analysis of western blot for all the groups. ***P < 0.001, ****P < 0.0001. **G** IGF2BP2-overexpressed FaDu cells or corresponding control cells underwent transfection with si-NC or si-Slug for 48 h, and the expression of Slug, vimentin, and E-Cadherin were assessed by western blot analysis (left) and analyzed quantitatively (right). *P < 0.05, **P < 0.01, ***P < 0.001. **H** After transfection with si-NC or si-Slug for 48 h, the wound healing of IGF2BP2-overexpressed FaDu cells and corresponding control cells were recorded (left) and analyzed quantitatively (right). **P < 0.01. **I** Representative images showing high or low expression of Slug and IGF2BP2 in HNSCC tissues. Scale bars: 200 μm. **J** Correlation analysis between Slug and IGF2BP2 in HNSCC tissues (*n* = 78). All the data are presented in the form of mean ± SD from three independently performed experiments

Given that EMT is directly or indirectly moderated by multiple predominant transcription factors (TFs), such as Snail, Slug, ZEB1, and Twist [37, 38], we therefore searched for the possible TFs that are regulated by IGF2BP2. In the TCGA database for HNSCC, the spearman correlation analysis revealed the strongest positive correlation between Slug and IGF2BP2 in 502 HNSCC tissue samples compared to Snail, ZEB1, and Twist, indicating a potential positive regulatory mechanism between IGF2BP2 and Slug in HNSCC (Fig. 6A). Moreover, significantly decreased expression levels of Slug from IGF2BP2^high^-expressed HNSCC tissues to IGF2BP2^low^-expressed HNSCC tissues to normal adjacent tissues were observed based on the TCGA database (Fig. 6B). In order to clarify the regulatory relationship between IGF2BP2 and Slug, we evaluated the expression of Slug in HNSCC cells expressing different levels of IGF2BP2. RT-qPCR and western blot analyses revealed that IGF2BP2 inhibition significantly decreased Slug expression at mRNA as well as protein levels in FaDu and SCC15 cells, whereas overexpression of IGF2BP2 significantly enhanced Slug expression (Fig. 6C-F). Meanwhile, there were no significant or consistent changes in the mRNA levels of Snail, ZEB1, and Twist in FaDu and SCC15 cells (Fig. 6C). Although the roles of Slug in promoting EMT have been well studied [39, 43], we further investigated the roles of Slug in IGF2BP2-regulated EMT of cancer cells. The results of the western blot analysis indicated that Slug knockdown antagonized the upregulation of vimentin and downregulation of E-Cadherin induced by IGF2BP2 overexpression in FaDu cells (Fig. 6G). Conversely, IGF2BP2 overexpression partially reversed vimentin’s downregulation and E-Cadherin’s upregulation caused by Slug inhibition in FaDu cells (Fig. 6G). Furthermore, wound-healing assays showed that silencing Slug attenuated the promotion of cell migration via IGF2BP2 overexpression in FaDu cells (Fig. 6H). To evaluate the correlation between IGF2BP2 and Slug expression levels in HNSCC specimen, we performed IHC staining of Slug and IGF2BP2 in 78 cases of HNSCC specimens. Generally, IGF2BP2 high expression specimens were characterized by high Slug expression, whereas IGF2BP2 low expression specimens displayed low to moderate expression of Slug (Fig. 6I). Based on IHC quantification score, IGF2BP2 and Slug expression disclosed a robust positive correlation (*r* = 0.752, *p*-value < 0.001) (Fig. 6J). Collectively, these findings indicate that Slug has a pivotal role in the IGF2BP2-regulated EMT in HNSCC.

### IGF2BP2 regulates slug mRNA stability via m6A modification

**Fig. 7:**
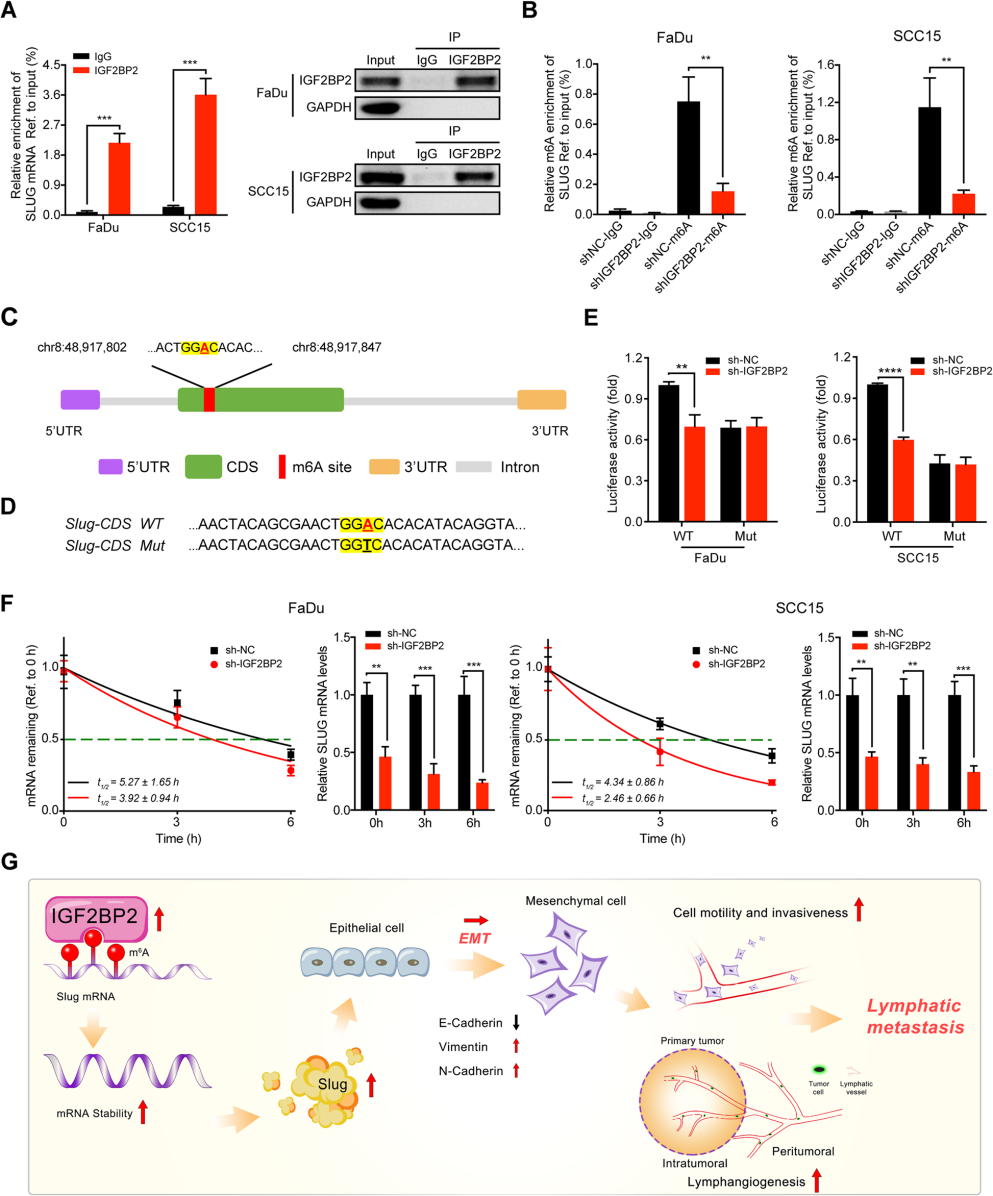
IGF2BP2 regulates slug mRNA stability via m6A modification. **A** RIP-qPCR analysis showing the enrichment of Slug mRNA in anti-IGF2BP2 precipitates (left panel). Western blot detected the IGF2BP2 immunoprecipitation efficiency of IGF2BP2 RIP assay in FaDu and SCC15 cells (right panel). GAPDH and IgG served as an internal or negative control, respectively. ***P < 0.001. **B** MeRIP-qPCR analysis showing the m6A enrichment of Slug mRNA using anti-IgG and anti-m6A antibodies in FaDu and SCC15 cells after silencing IGF2BP2. **P < 0.01. **C** A very high-confidence m6A site was identified in the CDS region upon Slug mRNA based on the SRAMP software analysis. **D** Schematic representation of Slug- WT (wild-type) or Slug- MT (mutated-type) sequence. **E** Luciferase reporter assays measured the luciferase activities of Slug-CDS WT or Slug-CDS Mut in FaDu and SCC15 cells with IGF2BP2 knockdown. **P < 0.01, ****P < 0.0001. **F** After silencing IGF2BP2 in FaDu and SCC15 cells, the mRNA half-lives and expression of Slug were analyzed at the predetermined times following actinomycin D (5 μg/ml) treatment. **G** A graphic illustration of the proposed mechanism in this study. In brief, IGF2BP2 was highly expressed in HNSCC patients, and recognized and bound to the m6A site upon Slug mRNA to maintain its mRNA stability and expression, thereby promoting cell migration and invasion through EMT progress, and leading to the lymphatic metastasis in HNSCC. **P < 0.01, ***P < 0.001, ****P < 0.0001. All the data are presented in the form of mean ± SD from three independently performed experiments

Since IGF2BP2 is considered to be an RNA binding protein, also known as “reader” in m6A RNA modification [8, 9, 19], we investigated whether IGF2BP2 interacts with Slug via m6A modification. First, we performed RIP-qPCR assays using the anti-IGF2BP2 antibody in FaDu and SCC15 cells, and the results showed significant enrichment of Slug mRNA compared to the IgG control group, confirming the interaction between IGF2BP2 and Slug mRNA (Fig. 7A). In order to verify whether Slug is affected by m6A modification, leading to the recognition of the methylated Slug by IGF2BP2, we conducted MeRIP-qPCR assays and determined that knockdown of IGF2BP2 markedly decreased the m6A levels of Slug in FaDu and SCC15 cells compared with the corresponding control cells (Fig. 7B). Based on the SRAMP software analysis, we identified a very high-confidence m6A site in the CDS region upon Slug mRNA (Fig. 7C). To validate the putative m6A site, we performed luciferase reporter assays using a luciferase reporter containing a wild-type (WT) Slug-CDS or mutated-type (MT) Slug-CDS sequence (GAACU to GACCU) (Fig. 7D). As expected, the luciferase activity was significantly attenuated in Slug-WT when IGF2BP2 was silenced, while that of Slug-MT seemed to be unaffected (Fig. 7E). Furthermore, mRNA stability assays revealed that the mRNA expression of Slug was decreased and the mRNA half-lives of Slug were continually reduced by IGF2BP2 silencing in FaDu and SCC15 cells (Fig. 7F). In general, these findings suggest that IGF2BP2 directly binds the CDS region upon Slug mRNA in an m6A modification-dependent manner and stabilizes its mRNA.

## Discussion

The primary cause of a poor prognosis in HNSCC patients is LN metastasis [2, 48]. Although surgery is a reliable treatment in localized tumor in HNSCC [4], treatment options for metastatic HNSCC are currently limited. Thus, it is of utmost importance to investigate the potential molecular mechanisms involved in lymphatic metastasis, and to identify novel and efficient targets for therapeutic strategies. In this study, we identified IGF2BP2 as a potential prognostic marker in HNSCC. Moreover, IGF2BP2 overexpression in HNSCC cells was required in multiple steps of the lymphatic metastatic process, mainly including enhanced ability of cell motility and invasiveness, and formation of lymphatic vessels. In addition, IGF2BP2 regulated Slug expression, a key transcriptional factor of EMT, to facilitate the EMT program and metastasis of HNSCC cells. More importantly, we found that IGF2BP2 directly interacted with Slug mRNA and promoted its mRNA stability in an m6A-dependent manner (Fig. 7G). These results offer novel insights related to the molecular mechanisms of cancer metastasis.

IGF2BP2, which acts as a novel m6A reader, is related to different biological processes, for instance, cell self-renewal, stemness maintenance, proliferation, and metastasis [10–12]. Previous studies have reported that IGF2BP2 is amplified and overexpressed in multiple cancers, and closely associated with clinical characteristics and poor prognosis [15, 52, 53]. The findings in this study have shown a high expression of IGF2BP2 in HNSCC tissues, and higher IGF2BP2 expression was significantly correlated to pathological N classification, LN metastasis and poor OS probability, indicating that IGF2BP2 could function as a prognostic biomarker in HNSCC, and plays a potential role in lymphatic metastasis of HNSCC. M6A modification has been reported to be associated with lymphatic metastasis in different cancers. Guo et al. [30] have found that the expression of the m6A reader HNRNPA2B1 is positively correlated with lymphatic metastasis, and promotes cell proliferation, migration, and invasion of ESCA. Wang et al. [31] have demonstrated that METTL3, a m6A writer, is closely associated with LN metastasis and a poor prognosis in cervical cancer. Nonetheless, which role IGF2BP2 plays in LN metastasis of HNSCC is still unknown. In the present study, it was shown that IGF2BP2 was highly expressed in HNSCC tissues with lymphatic metastasis compared with those without lymphatic metastasis, implying a potential role of IGF2BP2 in promoting lymphatic metastasis in cancers. To verify this, a popliteal LN metastasis model was constructed by injecting HNSCC cells into the footpad of nude mice, which is a sensitive and quantitative method of assessing lymphatic metastasis in vivo [54]. We found that IGF2BP2 knockdown could significantly inhibit lymphatic metastasis in vivo. Lymphangiogenesis is a rate-limiting step for the LN metastasis of cancer [55]. Consistently, we observed that silencing IGF2BP2 suppressed the formation of microlymphatic vessels in both the intratumoral and peritumoral regions of mouse tissues. Collectively, this study is the first to report on the discovery of the oncogenic role of IGF2BP2 in promoting lymphatic metastasis of HNSCC, indicating the broadly clinical significance of IGF2BP2 in cancer metastasis.

During the LN metastasis process, one important step is that tumor cells gain the capability of migration and invasion, and then move toward the lymphatic capillaries [48, 49]. It has been established that EMT is a crucial step in metastasis and invasion of multiple cancers [35]. In this study, we found that IGF2BP2 could promote the EMT program to increase the mobility and invasiveness of HNSCC cells in vitro, which is in accordance with other studies [52, 56]. The EMT process is controlled by a series of EMT-TFs, whose expression and importance are tissue-specific [37, 38]. Interestingly, among the EMT-TFs, such as Snail, Slug, ZEB1, and Twist, only Slug shows the strongest correlation with IGF2BP2 and is regulated by IGF2BP2 in HNSCC cell lines, thereby promoting the EMT program and cell metastasis. Slug, a conserved TF, plays an essential role in EMT during cancer metastasis [39, 40]. Puram et al. [57] investigated the intertumoral heterogeneity and metastasis between primary HNSCC tumors and matched LNs using single-cell transcriptomic analysis, and they found that Slug was the only EMT-related TF detected in HNSCC cells and closely correlated with the program across tumors. These discoveries align with our findings, further supporting the oncogenic role of IGF2BP2 in lymphatic metastasis of HNSCC cells.

The biological importance of m6A modification is determined by m6A readers that can recognize and directly bind to the m6A modification sites upon its target mRNAs, thus affecting their fate [20, 21]. To further investigate the regulatory mechanism between IGF2BP2 and Slug, we conducted RIP-qPCR analysis and determined that Slug is the direct target mRNA of IGF2BP2. Subsequently, MeRIP-qPCR analysis verified that IGF2BP2 regulated the mRNA expression of Slug through m6A modification. Furthermore, we predicted a very high-confidence m6A site in the CDS region upon Slug mRNA based on the SRAMP software analysis, which is a reliable and classic sequence-based m6A site predictor [58]. The results of the luciferase reporter analysis also confirmed this prediction. M6A readers have been linked to a variety of cellular processes, such as mRNA stability [23, 24, 26]. The Actinomycin D experiment confirmed that IGF2BP2 knockdown could significantly inhibit the mRNA stability of Slug. Similarly, Li et al. [59] demonstrated that IGF2BP2 can recognize and combine with the RNA methylation modification written by METTL3 to inhibit the degradation of SOX2, thereby promoting tumor progression. Hou et al. [60] revealed that IGF2BP2 cooperating with DHX9 enhances the HMGA1 mRNA stability by binding its 3’UTR and facilitates colorectal cancer proliferation and metastasis. Overall, these findings demonstrated that IGF2BP2 directly binds to the m6A site of the CDS region upon Slug mRNA and promotes the mRNA stability in a m6A-dependent manner, thereby facilitating EMT and lymphatic metastasis in HNSCC.

There are also some limitations in this study. First, the overall number of specimens is limited because access to tissue is invariably difficult. Second, although our findings reveal the oncogenic role of IGF2BP2 in promoting the lymphatic metastasis and EMT process, the regulatory mechanism between LN metastasis and EMT is not yet fully explained. This is probably because cancer cells undergoing EMT acquire the capacity to hijack a variety of chemotactic signals and apply them to disseminate through the lymphatic system [61]. However, a clearer understanding of the detailed mechanisms requires further investigation.

## Conclusions

In summary, our study reveals for the first time the clinical and biological function of IGF2BP2 in facilitating lymphatic metastasis in HNSCC, and demonstrated that IGF2BP2 promotes EMT and cell metastasis via stabilizing Slug mRNA in an m6A dependent-manner. These outcomes indicate that IGF2BP2 could function as a predictive biomarker of LN metastasis and potential target for anti-metastasis therapies for HNSCC patients.

## Supplementary Information

**Additional file 1.**

**Additional file 2.**

## Data Availability

All data analyzed in this study are available from the corresponding author upon reasonable request.

## Abbreviations

HNSCC: Head and neck squamous carcinoma cells
LN: Lymph node
IGF2BP2: Insulin-like growth factor 2 mRNA-binding protein 2
RBP: RNA binding protein
m6A: N6-methyladenosine
YTHDF: YT521-B homology domain family
IGF2BPs: Insulin-like growth factor 2 mRNA-binding proteins
EMT: Epithelial-mesenchymal transition
EMT-TFs: EMT transcriptional factors
CDS: Coding sequence
TCGA: The Cancer Genome Atlas
HR: Hazard ratio
CI: Confidence interval
IHC: Immunohistochemistry
siRNA: Small interfering RNA
shRNA: Short hairpin RNA
MOI: Multiplicity of infection
qRT-PCR: Quantitative real-time PCR
RIP: RNA binding protein immunoprecipitation
MeRIP: Methylated RNA immunoprecipitation
OS: Overall survival
NAT: Normal adjacent tissue
MLD: Microlymphatic vessel density
WT: Wild-type
MT: Mutated-type
T: Tumor
LM (+): Lymphatic metastasis
LM (−): Non-lymphatic metastasis
