# Supplementary material for: RNA N6-methyladenosine reader IGF2BP2 promotes lymphatic metastasis and epithelial-mesenchymal transition of head and neck squamous carcinoma cells via stabilizing slug mRNA in an m6A-dependent manner
Source: J Exp Clin Cancer Res. 2022 Jan 3;41:6. doi: 10.1186/s13046-021-02212-1 (PMC8722037; doi:10.1186/s13046-021-02212-1)
Supplement: Supplementary file 1 — Additional file 1. [file 13046_2021_2212_MOESM1_ESM.docx]

**Table S1. Primers used in the experiments.**

| **Gene name** | **Primer sequence** | | **Application** |
| --- | --- | --- | --- |
| IGF2BP2 | F | 5′-AGTGGAATTGCATGGGAAAATCA-3′ | qRT-PCR |
|  | R | 5′-CAACGGCGGTTTCTGTGTC-3′ |  |
| E-Cadherin | F  R | 5′-ATTTTTCCCTCGACACCCGAT-3′  5′-TCCCAGGCGTAGACCAAGA-3′ | qRT-PCR |
| N-Cadherin | F | 5’-TGCGGTACAGTGTAACTGGG-3′ | qRT-PCR |
|  | R | 5’-GAAACCGGGCTATCTGCTCG-3′ |  |
| Vimentin | F  R | 5’-AGTCCACTGAGTACCGGAGAC-3′  5’-CATTTCACGCATCTGGCGTTC-3′ | qRT-PCR |
| Snail | F  R | 5’-TCGGAAGCCTAACTACAGCGA-3′  5’-AGATGAGCATTGGCAGCGAG-3′ | qRT-PCR |
| Slug | F  R | 5’-TGTGACAAGGAATATGTGAGCC-3′  5’-TGAGCCCTCAGATTTGACCTG-3′ | qRT-PCR/RIP-qPCR/ MeRIP-qPCR |
| ZEB1 | F | 5’-CAGCTTGATACCTGTGAATGGG-3′ | qRT-PCR |
|  | R | 5’-TATCTGTGGTCGTGTGGGACT-3′ |  |
| Twist | F  R | 5’-GTCCGCAGTCTTACGAGGAG-3′  5’-GCTTGAGGGTCTGAATCTTGCT-3′ | qRT-PCR |
| GAPDH | F  R | 5’-CAGCGACACCCACTCCTC-3′  5’-TGAGGTCCACCACCCTGT-3′ | qRT-PCR |
| si-NC | sense  anti-sense | 5’-UUCUCCGAACGUGUCACGUTT-3′  5’-ACGUGACACGUUCGGAGAATT-3′ | si-RNA |
| si-IGF2BP2#1 | sense  anti-sense | 5'-GCGAAAGGAUGGUCAUCAUTT-3'  5'-AUGAUGACCAUCCUUUCGCTT-3' | si-RNA |
| si-IGF2BP2#2 | sense  anti-sense | 5'-GCUGUUAACCAACAAGCCATT-3'  5'-UGGCUUGUUGGUUAACAGCTT-3' | si-RNA |
| si-IGF2BP2#3 | sense  anti-sense | 5'-ACAGGACUGUCCGUGCUAUTT-3'  5'-AUAGCACGGACAGUCCUGUTT-3' | si-RNA |
| si-Slug | sense  anti-sense | 5'-CAUAGGAAGAGAUCUGCCAUU-3'  5'-UUGUAUCCUUCUCUAGACGGU-3' | si-RNA |
| sh-NC |  | 5′-UUCUCCGAACGUGUCACGU-3' | sh-RNA |
| sh-IGF2BP2 |  | 5'-GCGAAAGGAUGGUCAUCAUTT-3’ | sh-RNA |

Abbreviations: F: Forward; R: Reverse; qRT-PCR: quantitative real-time PCR; RIP: RNA binding protein immunoprecipitation; MeRIP: Methylated RNA immunoprecipitation; si-RNA: Small interfering-RNA; sh-RNA: Short hairpin-RNA
