# Supplementary material for: RNA N6-methyladenosine reader IGF2BP2 promotes lymphatic metastasis and epithelial-mesenchymal transition of head and neck squamous carcinoma cells via stabilizing slug mRNA in an m6A-dependent manner
Source: J Exp Clin Cancer Res. 2022 Jan 3;41:6. doi: 10.1186/s13046-021-02212-1 (PMC8722037; doi:10.1186/s13046-021-02212-1)
Supplement: Supplementary file 2 — Additional file 2. [file 13046_2021_2212_MOESM2_ESM.docx]

**Table S1. Antibodies used in the experiments.**

| **Antibody** | **Source** | **No. of Catalogue** | **Dilution** | | |
| --- | --- | --- | --- | --- | --- |
|  |  |  | **WB** | **IHC** | **IF** |
| IGF2BP2 | Abcam, USA | ab124930 | 1:2000 | 1:100 |  |
| E-Cadherin | Cell Signaling Technology, USA | 24E10 | 1:1000 |  | 1:200 |
| N-Cadherin | Cell Signaling Technology, USA | D4R1H | 1:1000 |  |  |
| Vimentin | Cell Signaling Technology, USA | D21H3 | 1:1000 |  | 1:100 |
| Slug | Cell Signaling Technology, USA | C19G7 | 1:1000 |  |  |
| LYVE-1 | Abcam, USA | ab218535 |  | 1:100 |  |
| GAPDH | Proteintech, China | 10494-1-AP | 1:3000 |  |  |

Abbreviations: WB: Western blot; IHC: Immunohistochemistry; IF: Immunofluorescence
